# Development of Compact Electronics for QEPAS Sensors

**DOI:** 10.3390/s25216718

**Published:** 2025-11-03

**Authors:** Vincenzina Zecchino, Luigi Lombardi, Cristoforo Marzocca, Pietro Patimisco, Angelo Sampaolo, Vincenzo Luigi Spagnolo

**Affiliations:** 1 PolySense Lab, Inter-University Department of Physics, University and Polytechnic University of Bari, 70126 Bari, Italy; v.zecchino@phd.poliba.it (V.Z.); pietro.patimisco@uniba.it (P.P.); angelo.sampaolo@poliba.it (A.S.); vincenzoluigi.spagnolo@poliba.it (V.L.S.); 2 Department of Electrical and Information Engineering, Polytechnic University of Bari, 70125 Bari, Italy; 3 PolySenSe Innovations S.R.L., 70126 Bari, Italy; luigi.lombardi@polysense.it

**Keywords:** QEPAS, quartz tuning fork, front-end electronics, lock-in amplifier, function generation

## Abstract

**Highlights:**

**What are the main findings?**

- Compact electronics for QEPAS sensors, featuring a fully differential preamplifier, a digital function generator, and an FPGA-based lock-in amplifier, replaces bulky lab instruments.
- Clear H_2_O and CO_2_ detection achieved with low-power laser sources.

**What is the implication of the main finding?**

- Compact, low-cost electronics enables the development of portable QEPAS sensors.
- The reduced power consumption and size of the proposed electronics expand the range of QEPAS applications.

**Abstract:**

Remarkable advances in Quartz-Enhanced Photoacoustic Spectroscopy (QEPAS) made it one of the most effective gas-sensing techniques in terms of sensitivity and selectivity. Consequently, its range of possible applications is continuously expanding, but in some cases is still limited by the cost and/or size of the equipment needed to im-plement a complete QEPAS sensor. In particular, bulky and expensive lab instruments are often used to realize the electronic building blocks required by this technique, which prevents, for instance, integration of the system on board a drone. This work addresses this issue by presenting the development of compact electronic modules for a QEPAS sensor. A very low-noise, fully differential preamplifier for the quartz tuning fork, with digital output and programmable gain, has been designed and realized. A compact FPGA board hosts both an accurate function generation module, which synthesizes the signals needed to modulate the laser source, and an innovative lock-in amplifier based on the CORDIC algorithm. QEPAS sensors based on the designed electronics have been used for the detection of H_2_O and CO_2_ in ambient air, proving the full functionality of all the blocks. These results highlight the potential of compact electronics to promote portable and cost-effective QEPAS applications.

## 1. Introduction

Quartz-Enhanced Photoacoustic Spectroscopy (QEPAS) is a highly sensitive technique for trace gas detection, which exploits the photoacoustic effect in combination with the resonant properties of quartz tuning forks (QTFs) [1,2,3]. In QEPAS, a laser beam, tuned to a wavelength corresponding to an absorption line of the target molecule, passes through a gas sample. The beam intensity is modulated sinusoidally, resulting in a periodic absorption of the radiation by the molecules. This periodic absorption induces localized heating and cooling of the gas sample, which in turn generates acoustic pressure waves at the laser modulation frequency.

The acoustic waves generated in the gas sample are detected by the quartz tuning fork (QTF), an electromechanical resonator characterized by narrow bandwidth and high quality factor. Due to the piezoelectric effect, the QTF converts these mechanical oscillations into a weak electrical signal. This signal is then amplified by a low-noise preamplifier and demodulated through a lock-in amplifier (LIA), which multiplies the QTF signal with a reference signal at the same frequency used for laser modulation or one of its harmonics and applies a narrow low-pass filter to the resulting signal, obtaining a DC component proportional to the target gas concentration, which is the desired output of the sensor [4].

Traditional QEPAS systems are based on benchtop instrumentations, such as lock-in amplifiers and function generators, that are bulky and unsuitable for compact or portable implementations. The present study focuses on the design and integration of compact electronic subsystems needed for the generation of the modulation signal, for the amplification of QTF signal, and for its demodulation. The development effort was directed toward a high-performance analog front-end and optimized digital architectures, excluding the circuitry related to laser control, such as the driver and temperature regulator, that will be implemented in the next steps of the research activity.

Thus, a compact electronic chain for the QEPAS system was implemented based on a variable gain, fully differential preamplifier for the QTF, specifically designed to enhance the signal-to-noise ratio (SNR) while minimizing susceptibility to common-mode noise and external electromagnetic interference. The amplified signal is digitized and then processed within a Field-Programmable Gate Array (FPGA), which hosts a fully digital lock-in amplifier for demodulation. A digital function generation module is also integrated on board the FPGA, which generates the samples of both the sine wave signals needed for laser modulation and used as LIA reference, respectively. The former is converted into analog form by means of a digital-to-analog converter (DAC) and used to modulate the laser current through a voltage-controlled current source.

Both the demodulation stage and the function generation module inside the FPGA leverage in an effective way CORDIC-based (COordinate Rotation DIgital Computer) algorithms, which enable efficient extraction of the amplitude and phase of the signal without the use of hardware multipliers. This approach improves computational efficiency and optimizes FPGA resource usage.

In summary, the compact electronics developed for a QEPAS system is composed of the following functional blocks: a fully differential preamplifier integrated on a small-size board that also includes resources for analog/digital conversion, an FPGA board hosting the function generator and digital lock-in amplifier, and a digital-to-analog (DA) board, which converts the digital modulation signal, generated by the FPGA, into an analog signal in order to send it to the laser driver. This board also hosts a microcontroller for system management and control.

**Figure 1:**
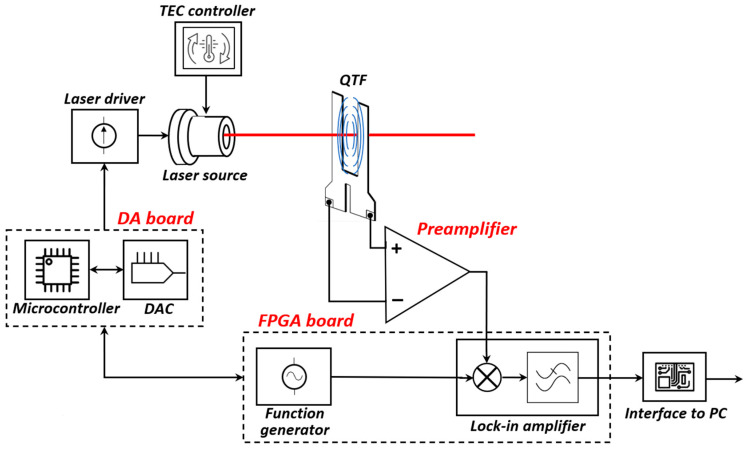
Main building blocks of a QEPAS sensor. The developed electronic blocks are highlighted in red.

A QEPAS sensor equipped with the designed circuits was assembled, according to the scheme of Figure 1, and was successfully used to prove the effectiveness of the electronic building blocks integrated into the application.

By replacing conventional analog equipment with digital signal generation and processing directly embedded in an FPGA, and by introducing a custom-designed analog front-end, the system achieves significant advantages in terms of SNR, compactness, power efficiency, cost and flexibility, thereby enabling future integration into portable QEPAS systems, also easily deployable on board a drone. This paves the way for the exploitation of the QEPAS sensing technique in a broader range of applications, for instance, in the field of environmental monitoring and breath analysis for medical diagnostics.

This paper is organized as follows. Section 2 presents the design of the fully differential preamplifier board, providing a detailed analysis of its individual stages as well as the integrated DAC and ADC components. Section 3 addresses the implementation of the digital function generator and the digital lock-in amplifier, with a particular focus on the CORDIC-based demodulation scheme. It also describes the design and integration of the DA board with the microcontroller unit. Section 4 presents the experimental validation of the system, including performance measurements and characterization results. Finally, Section 5 summarizes the key findings and discusses possible directions for future research and development.

## 2. Front-End Electronics for the Quartz Tuning Fork

The preamplifier board is designed to read out and amplify the signal generated by the QTF in the QEPAS system [5]. The most common front-end configuration is the transimpedance preamplifier (TIA) [6]. This solution provides a simple way of converting the current signal generated by the QTF into a measurable voltage [5,6,7,8]. However, its noise performance is typically limited by the feedback resistor, whose thermal noise contributes significantly to the input current noise. Alternative front-end topologies have been proposed to improve sensitivity, such as voltage amplifiers, which also implemented in fully differential configurations to reduce common-mode interference [9], and to charge amplifiers [7]. Among these, the charge amplifier solution exhibits the lowest input equivalent current noise thanks to the use of capacitive feedback instead of the noisy resistive one of a TIA, and the absence of bias resistors at the input stage, which represent an additional noise source in voltage amplifiers.

The goal of our design is to overcome the limitations of conventional single-stage solutions. The primary focus during the preamplifier development was therefore the definition of a circuit architecture capable of efficiently interfacing with the sensor while minimizing susceptibility to external electromagnetic interference using a fully differential structure. A further requirement for the circuit was flexibility, accommodating different operating conditions and target gas concentrations through simple gain adjustment. To meet these specifications, a two-stage, fully differential front-end architecture with variable gain was adopted.

**Figure 2:**
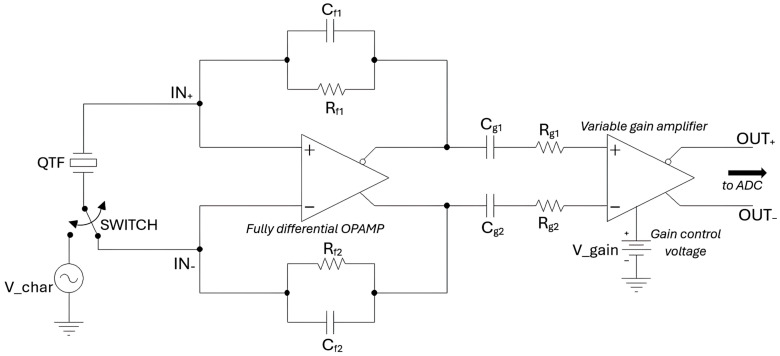
Simplified schematic of the QTF preamplifier.

A simplified schematic of the preamplifier is shown in Figure 2.

The first stage is based on a fully differential operational amplifier (OPAMP), which can be configured either as a transimpedance preamplifier, with typical values of R_f1_ = R_f2_ = 10 MΩ and C_f1_ and C_f2_ not placed, or as a charge preamplifier, using large values for R_f1_ and R_f2_, for instance, 100 MΩ, and applying a suitable amount of capacitive feedback C_f1_ = C_f2_. This configurability provides additional flexibility to the system, making it suitable for different cases and applications.

The QTF is DC-connected directly to the inputs of the first stage during the QEPAS operating mode of the sensor, or it can be connected directly to an external sinusoidal input stimulus with variable frequency V_char, by means of a digitally controlled switch, during the QTF characterization process. In characterization mode, the QTF generates a sinusoidal current in response to the external excitation, and this signal can be used to determine the resonant frequency of the sensor, identifying the frequency corresponding to the maximum amplitude response. This same frequency, or a sub-harmonic, is subsequently employed to modulate the laser in QEPAS operating mode, thus ensuring excitation of the QTF at its resonance and maximizing the signal level at the sensor output [10].

**Figure 3:**
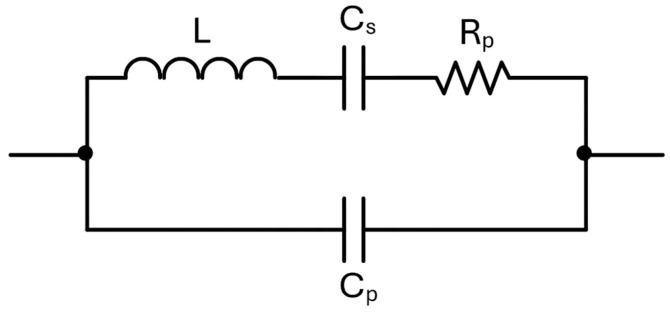
Butterworth–Van Dyke model of the QTF.

In particular, the first stage of the preamplifier employs the THS4567 as fully differential OPAMP, powered by a single 5 V supply. The DC input bias currents of the OPAMP, which can be much greater than the amplitude of the QTF signal, can only cause small deviations of its common-mode output voltage and cannot give contributions to the differential output. Moreover, the input offset current of the OPAMP can cause a non-zero DC differential output, but this is removed by AC coupling the second stage, by means of the capacitors C_g1_ and C_g2_. The terminals of the QTF, in QEPAS operating mode, are kept in virtual short circuit by the OPAMP at a constant input common-mode voltage V_ICM_ ≅ 1.5 V internally set by the OPAMP. This allows getting rid of any influence of the parasitic capacitance C_p_ seen between the terminals of the QTF in the Butterworth–Van Dyke model, shown in Figure 3 [11].

When the stage is configured as a charge amplifier, its transimpedance gain $A_{R}$ can be expressed as

$$A_{R}=\frac{2}{2\pi f_{0}C_{f}},$$

where $f_{0}\;$ is the target operating frequency, i.e., the resonant frequency of the employed QTF. The feedback resistors are very large, so their contribution to the equivalent input current noise is negligible. They are needed only for setting correctly the operating point of the OPAMP. With this arrangement, the dominant contribution to the total output noise of the circuit is given by the resistor R_p_ of the QTF model. Moreover, the fully differential architecture eliminates any contribution of common-mode interferences to the output signal.

The second stage of the circuit is a variable gain amplifier (VGA), also fully differential. This stage is based on the AD8338 VGA, also powered by a 5 V single supply. The voltage gain $A_{V}$ of the AD8338 is controlled by the DC level V_gain applied to its gain control input, allowing linear adjustment from 0 dB to 80 dB as V_gain varies between 0.1 V and 1.1 V. For instance, the overall transimpedance gain of the preamplifier with the first stage configured as a charge amplifier $A_{Rtot}=A_{R}\cdot A_{V}$, choosing $C_{f}=C_{f1}=C_{f2}=2.2\;pF$, ranges approximately from 11.7 MΩ (for V_gain = 0.1 V) up to 11.7 × 10^4^ MΩ (for V_gain = 1.1 V) for a typical custom QTF with $f_{0}$ around 12.4 kHz [12].

Instead, when the first stage is configured as a transimpedance amplifier, since its gain $A_{R}$ is equal to $R_{f1}+R_{f2}$, setting $R_{f1}=R_{f2}=10\;M\Omega$, the overall transimpedance gain of the preamplifier can be varied from 20 MΩ to 200 GΩ.

To provide additional flexibility in gain control, zero-ohm resistors $R_{g1}$ and $R_{g2}\;$ are placed in series with the VGA inputs (Figure 2). These can be replaced to modify the effective gain range. In fact, the intrinsic VGA gain $A_{V}$ is modified by a factor of $0.5/(0.5+R_{g})$, with $R_{g}=R_{g1}=R_{g2}$ expressed in kΩ. If, for instance, we choose $R_{g}=2.5$ kΩ, we will obtain a gain of the VGA variable between −16 dB and +64 dB, and the overall gain of the preamplifier, configured as a charge amplifier as described above, will be variable between 2 MΩ and 2 × 10^4^ MΩ if V_gain varies from 0.1 V to 1.1 V. Thus, the possibility of inserting resistors of different values for $R_{g1}$ and $R_{g2}$ guarantees a further degree of flexibility, making possible the use of the preamplifier in an extensive range of different cases.

Furthermore, switching between “Characterization” and “QEPAS” operating modes is accomplished using the MAX4624 analog switch, from Analog Devices (the “SWITCH” in Figure 2).

As already pointed out, in the proposed electronics system for the QEPAS sensor, an FPGA was used to implement the LIA and the function generation module, which operate entirely in the digital domain. Thus, the preamplifier output must be converted into digital form for LIA demodulation, and to preserve signal integrity, a fully differential analog-to-digital converter was integrated in the preamplifier board. Moreover, the sinusoidal excitation signal needed for the identification of the QTF resonant frequency in characterization mode is generated by the FPGA board and must therefore be converted into analog, to be applied to the QTF. For this purpose, a DAC was integrated on the preamplifier board [6].

**Figure 4:**
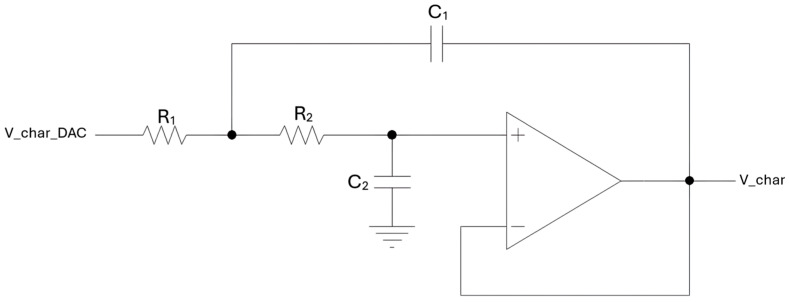
Low-pass Sallen–Key filter schematic: V_char_DAC is the output of the DAC on the preamp board, and V_char is the filtered signal applied to the QTF in characterization mode.

In addition, the output of the DAC, which converts into analog form the characterization sine wave, is processed through a second-order low-pass Sallen–Key filter, with a cut-off frequency of 50 kHz, also integrated on the preamplifier board. This smoothing filter is based on an AD8655 operational amplifier, from Analog Devices, and attenuates the high-frequency components of the DAC output signal, improving its spectral purity. The schematic of this filtering stage is shown in Figure 4 [13].

A further on-board DAC receives from the FPGA the digital word corresponding to the desired value of the VGA gain and generates the analog control voltage V_gain, enabling precise and programmable control of the overall gain of the QTF analog front-end chain.

To simplify the design and reduce the board size and complexity, AD and DA converters with serial digital interfaces were chosen so that communication with the FPGA take place over simple standard SPI interfaces. In particular, an ADC141S626 and two MAX5214 DAC were used so that the resolution of both digital inputs and output of the preamplifier board is 14 bits. The sampling period of both converters is $T_{S}=4.38\;\mu s$, corresponding to a sampling frequency $f_{s}\;$ of about 230 kS/s.

**Figure 5:**
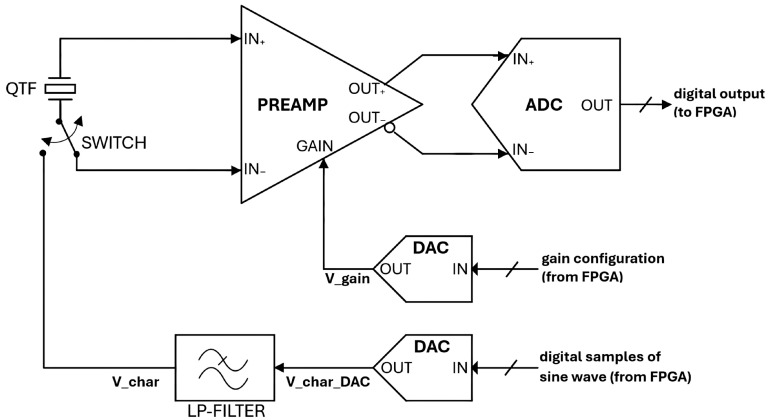
Block diagram of the QTF preamplifier board with A/D and D/A resources on board.

A simplified block diagram of the QTF preamplifier board is shown in Figure 5.

One of the most critical aspects in ensuring signal integrity on a mixed-signal board is the effective distribution and isolation of power supplies between analog and digital sections. In our design, separate reference levels were established for the ADC and DACs, each generated by dedicated integrated circuits. Furthermore, the board was fabricated with a four-layer stack-up, featuring continuous ground planes in the two internal layers. Moreover, careful decoupling and filtering of both the 3.3 V digital and 5 V analog power supplies were implemented to minimize interference between analog and digital domains.

**Figure 6:**
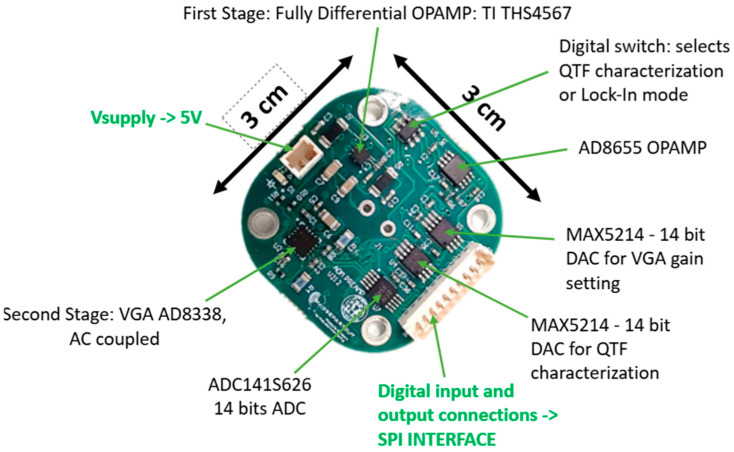
Picture of the preamplifier board.

Figure 6 is a picture of the preamplifier board prototype. The connectors and all the components are clearly visible, as well as the pads for the terminals of the QTF at the center of the board. The size of the board (3 cm × 3 cm) allows its integration in the acoustic detection module (ADM) [14], i.e., the small chamber with controlled temperature and pressure where the interaction between the laser beam and the target gas takes place, so that the interconnections between the terminals of the QTF and the preamplifier can be made as short as possible.

## 3. Function Generation Unit and Lock-In Amplifier

Both the lock-in amplifier and the function generation module are digital blocks synthesized on an FPGA device.

Lock-in amplifiers (LIAs) are widely used instruments for extracting weak signals from noisy environments through a demodulation process, which isolates the signal components that are coherent with a reference. This can be implemented using analog or digital solutions. Analog LIAs employ analog hardware multipliers for the convolution process, which are costly and complex when high precision is required, and analog filters, also very complex if the requirements in terms of selectivity are remarkably harsh. Digital LIAs, on the other hand, use digital multiplier modules that are cheaper and whose precision depends on the number of bits used for the digital representation of the signals. Digital filters can be easily made more selective and accurate than the analog counterparts.

In FPGA-based LIAs, the reference signal is typically generated using look-up tables (LUTs) containing sampled values of a sine wave, while demodulation relies on hardware multipliers [15]. These approaches demand significant memory and hardware resources, which cannot then be allocated to additional processing stages, such as filtering, to further reduce noise and improve overall performance.

To overcome these constraints, we propose an FPGA-based LIA architecture that employs the COordinate Rotation DIgital Computer (CORDIC) algorithm for both reference generation and demodulation. This approach replaces traditional multipliers with simple shift operations, drastically reducing hardware utilization while maintaining accuracy and performance. To the best of our knowledge, no previous FPGA-based LIA design has been based on CORDIC for both the reference generation and demodulation stages.

A compact and low-cost DE0-Nano FPGA development board from Terasic, Taiwan, based on the INTEL Cyclone IV EP4CE22 device, was selected for the implementation of these building blocks. In addition, to complete the function generation unit, a custom DA board, assembled on top of the FPGA board, hosts the DAC, which converts the digital modulation signal into the analog waveform applied to the input of the laser driver. This board also integrates a microcontroller, which provides system management functions.

A detailed description of the designed modules follows.

### 3.1. Function Generation Module

The purpose of this module is the generation of the digital samples of all the signals needed to operate the QEPAS sensor.

**Figure 7:**
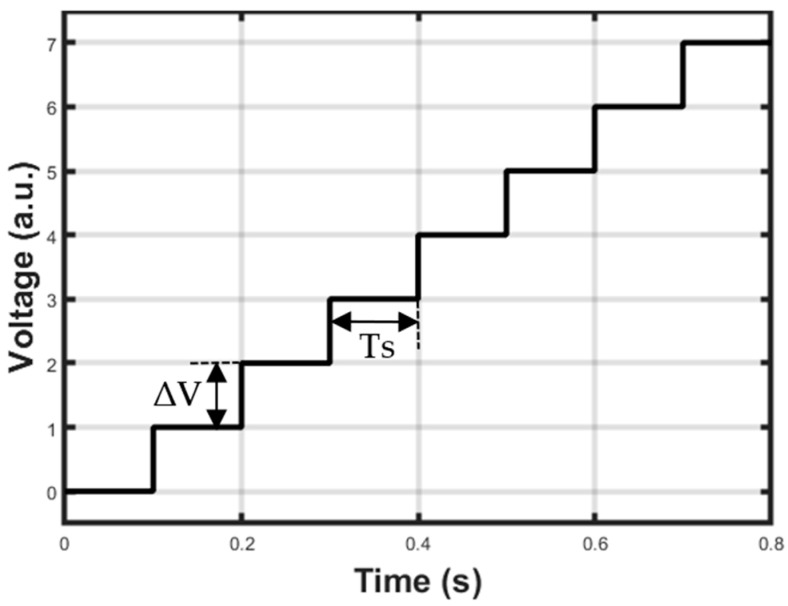
Example of staircase signal for scanning the laser wavelength around the absorption line of the target gas.

First of all, the laser diode must be fed with a current source controlled by a waveform generator. When the QEPAS sensor is operated in the so-called scan mode, the laser emission wavelength must be varied around the absorption line of the target gas in order to accurately identify its location. Therefore, the signal that controls the laser current is composed of two superimposed components: a staircase signal, as depicted in Figure 7, needed to scan the wavelength interval of interest, and a small amplitude sine wave, needed to modulate the laser intensity at the resonant frequency of the QTF or one of its sub-harmonics. The duration T_S_ of the single step must allow the completion of the lock-in operation and depends on the value selected for the integration time of the LIA. Both T_S_ and the amplitude ΔV of the single step have programmable values.

**Figure 8:**
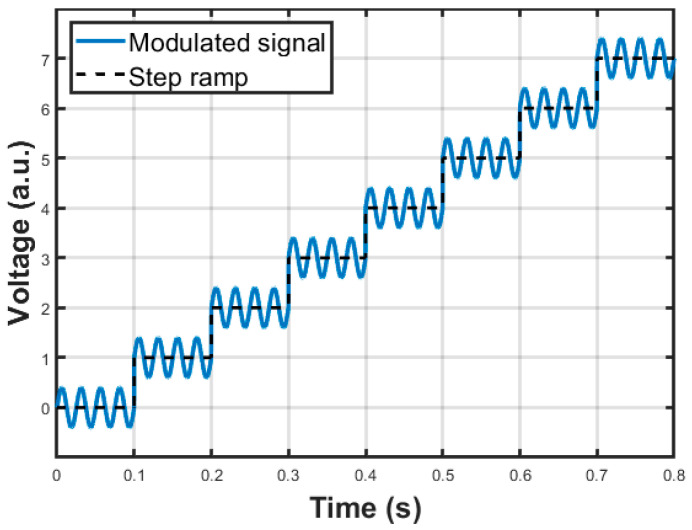
Example of signal waveform used to control the output current of the laser driver.

Concerning the modulation sine wave, a classic 2f demodulation scheme was adopted for the QEPAS sensor. In this approach, the modulation frequency of the laser source is $f_{0}/2$, where f0 is the resonant frequency of the QTF, whereas the demodulation frequency for the signal produced by the QTF, i.e., the reference frequency of the LIA, is equal to f0. Even though the 2f demodulation scheme in scan mode does not allow extracting the shape of the absorption line of the target gas without distortion, it is able to provide the peak of the signal in correspondence with the peak of the absorption line without errors. Moreover, the peak value is free from constant background contribution, and as a consequence, background compensation schemes are not needed [16,17]. Thus, the function generation module integrated in the FPGA outputs the samples of a sine wave signal with frequency $f_{0}/2$ and programmable amplitude so that it is possible to vary the modulation index and to adapt the signal amplitude to the laser driver of choice. The resolution of the sine wave samples is 14 bits, and the rate is about 165 kS/s. As mentioned, the modulation signal is added to the value of the staircase to generate a voltage signal represented in principle in Figure 8, to be used to control the output current of the laser driver.

As already pointed out, the resonant frequency of the QTF $f_{0}\;$ must be found by means of electrical characterization of the sensor. Thus, the function generation module also generates the samples of the sine wave applied to the QTF in the characterization phase (see Figure 5), scanning the frequencies around the resonance of the QTF. The span of the frequency scan, the number of frequency points, and the amplitude of the characterization signal are all programmable features. The resolution and the rate of the samples are the same as the laser modulation signal.

All the parameters of the staircase signal and of both modulation and characterization sine waves are configured by means of an external microcontroller, which communicates with the FPGA via a standard SPI interface.

The samples of both modulation and characterization sinusoidal signals are generated by exploitation of the well-known COordinate Rotation DIgital Computer (CORDIC) algorithm [18]. This algorithm is particularly suitable for digital hardware such as FPGAs and ASICs, as it computes the value of trigonometric functions using only binary shifts, additions, and subtractions, avoiding the need for hardware multipliers or large look-up tables.

CORDIC exploits a fundamental geometric property of the circle with a unity radius: any point on the circumference, at an angle θ from the *x*-axis, has coordinates x=cosθ and y=sinθ. Therefore, computing trigonometric functions can be reduced to determining the Cartesian coordinates of a rotated vector on the unit circle. The CORDIC algorithm exploits this property by performing a sequence of discrete and predefined vector rotations in the Cartesian plane. In more detail, starting from an initial vector aligned with the *x*-axis, it rotates this vector by a number of suitable positive or negative elementary angles until the accumulated rotation angle approximates the desired angle θ with the required accuracy.

Considering a vector $p_{\alpha }=[x_{\alpha },y_{\alpha }]=[\rho \cdot cos\alpha ,\;\rho \cdot sin\alpha ]$, if we rotate it by an angle ψ, the Cartesian coordinates of the rotated vector *p_β_* = [$x_{\beta }$,$y_{\beta }$] are the following ones:

(1) $$x_{\beta }=\rho \cdot \cos\left(\alpha +\psi \right)y_{\beta }=\rho \cdot \sin\left(\alpha +\psi \right)$$

By applying the sum formulas for sine and cosine, the following rotation formulas are obtained:

(2) $$x_{\beta }=\rho \left(\cos\alpha \cdot \cos\psi -\sin\alpha \cdot \sin\psi \right)=\cos\psi \left(x_{\alpha }-y_{\alpha }\cdot \tan\psi \right)\;y_{\beta }=\rho \left(\sin\alpha \cdot \cos\psi +\cos\alpha \cdot \sin\psi \right)=\cos\psi \left(y_{\alpha }+x_{\alpha }\cdot \tan\psi \right)$$

If we consider rotation angles equal to $\psi_{i}={tan}^{-1}(2^{-i})$, it is possible to obtain the coordinates of the rotated vector, scaled by a factor $\cos\psi_{i}$, through multiplications by $2^{-i}$. In binary arithmetic, this corresponds to a simple bit-shift operation. Thus, for a given angle θ, we can obtain approximate values of cosθ and sinθ, scaled by a constant factor *K*, starting from the vector $p_{0}=[1,0]$ and rotating it by angles equal to $\psi_{i}=\pm {tan}^{-1}(2^{-i})$, until the accumulated rotation reaches the value of θ within the desired accuracy [19].

Single CORDIC iterations are described by the following equations:

(3) $$\varphi_{i+1}=\varphi_{i}-\sigma_{i}{\cdot \psi }_{i}\;x_{i+1}=x_{i}-\sigma_{i}{\cdot y}_{i}{\cdot 2}^{-i}\;y_{i+1}=y_{i}+\sigma_{i}{\cdot x}_{i}{\cdot 2}^{-i}.$$

Here, $\varphi_{0}$ is the target angle θ and $\sigma_{i}=+1$ when $\varphi_{i}\geq 0$, $\sigma_{i\;}=-1$ when $\varphi_{i}<0$.

When the number of iterations *i* tends to infinity, we obtain:

(4) $$\underset{i\to \infty }{\lim}\varphi_{i}=0\;\;\;\underset{i\to \infty }{\lim}x_{i}=K\cos\theta \;\;\;\underset{i\to \infty }{\lim}y_{i}=K\sin\theta ,$$

where

(5) $$K=\prod_{i=0}^{\infty }\frac{1}{\cos\psi_{i}}=\prod_{i=0}^{\infty }\sqrt{1+{\left(\tan\psi_{i}\right)}^{2}}=\prod_{i=0}^{\infty }\sqrt{1+2^{-2i}}$$

is a constant factor, equal to 1.64676 [20], which can be easily taken into account when the parameters of the sine wave to be synthesized are passed to the system.

**Figure 9:**
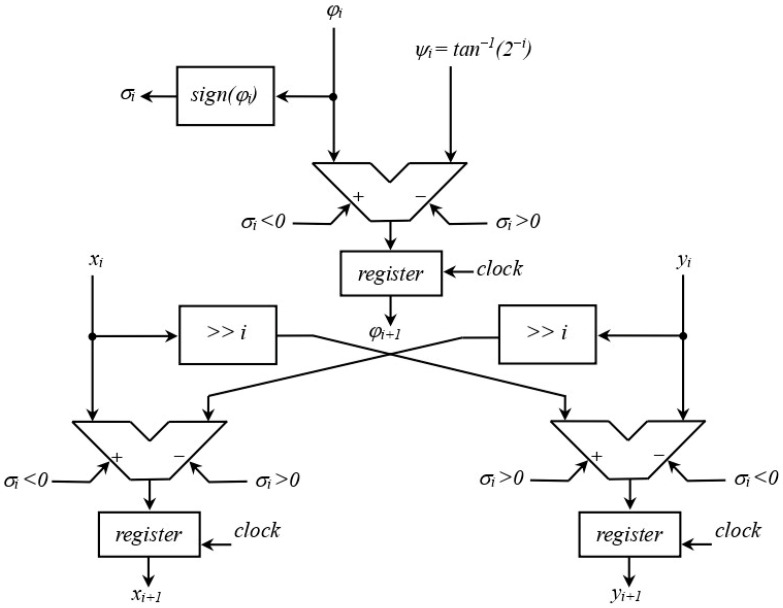
Hardware structure of the elementary cell which performs the single iteration of the CORDIC algorithm.

Figure 9 depicts the hardware structure of the elementary CORDIC cell which implements the single iteration represented by (3).

**Figure 10:**
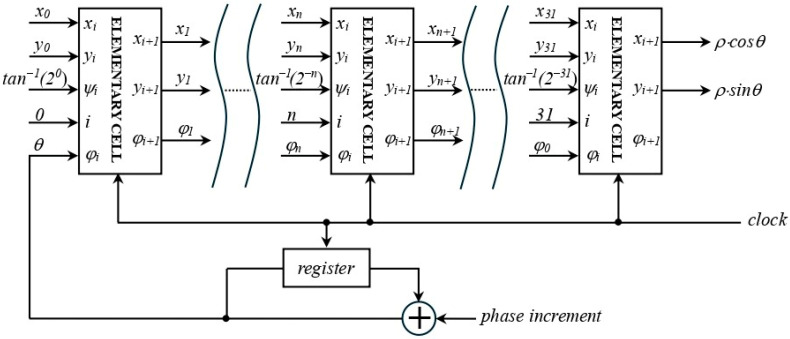
Block diagram of the CORDIC module.

The CORDIC module is composed of the pipeline of n=32 elementary cells, as represented in Figure 10. The frequency of the generated sine waves is decided by the value assigned to the phase increment and by the clock frequency $f_{ck}=50\;mHz$, with resolution equal to $\frac{f_{ck}}{2^{32}}≅11.6\;mHz$. All the digital signals are 32-bit words, and the values of the elementary rotation angles $\psi_{i}$ are stored in a very small look-up table. The accuracy of the digital sine wave samples depends on the phase noise of the clock signal, generated by a stable crystal oscillator, and on the intrinsic error of the CORDIC algorithm, which is very low for 32 iterations, of the order of $10^{-10}$.

The same CORDIC module is used to generate both the sine wave used to find the resonant frequency $f_{0}$ of the QTF, during the characterization phase, and the modulation signal for the laser current, with frequency $f_{0}/2$, during QEPAS operation.

### 3.2. DA Board

As previously mentioned, a dedicated board was designed to perform digital-to-analog conversion of the modulation signal for the laser driver. This board receives from the FPGA the digital samples of the sine wave at $f_{0}/2$ superimposed on the staircase signal, shown in Figure 8, and outputs the corresponding analog signals required to modulate the laser source. The core component is a DAC8164, a four-channel serial-input DAC. Three out of four channels are utilized so that it is possible to drive three different laser sources with the same board, in order to make possible the realization of a QEPAS sensor based on a multi-laser source for the detection of different target gases in sequence [21]. Each DAC output is passed through a second-order Sallen–Key low-pass biquad filter, with the same structure shown in Figure 4, to suppress high-frequency noise and obtain smooth analog waveforms.

The DA board is designed to allow direct connection to the DE0-Nano FPGA board via its dual 2 × 20 pin headers. Analog output signals are routed to the laser drivers through three SMA connectors. Additionally, the board acts as a mezzanine interface, routing signals between the FPGA board, the preamplifier board, and an external NUCLEO microcontroller board, from STMicroelectronics, used for exchanging data with a host PC during the experimental tests for the functional characterization of the electronic system. Moreover, an STM32 microcontroller, from STMicroelectronics, was also integrated on the DA board, with the purpose of allowing the DA board to serve not only as a digital-to-analog converter but also as the central control unit of the system, enhancing its compactness and simplifying the interconnections.

**Figure 11:**
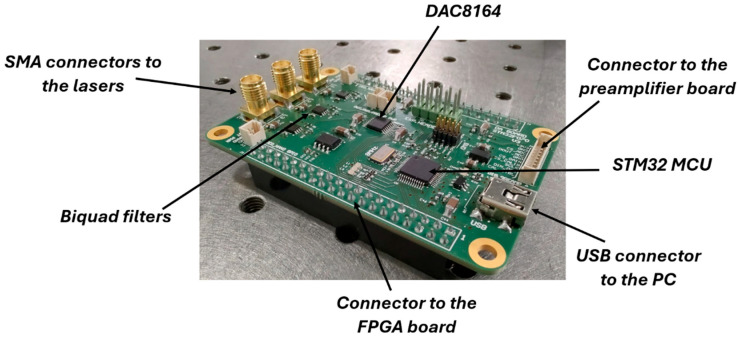
The prototype of the DA board.

The fabricated prototype of the DA board is shown in Figure 11. Its size is about 7.5 cm × 5 cm.

### 3.3. Lock-In Amplifier (LIA)

Synchronous detection by means of a lock-in amplifier (LIA) is used to extract weak signals, generated by low target gas concentrations, from noise [22]. As is well-known, an LIA multiplies the sinusoidal input $V_{in}sin(\omega_{0}t+\theta )$ with a reference signal at the same frequency $V_{ref}sin(\omega_{0}t)$, obtaining an output signal $V_{out}\;$ with difference and sum frequency components:

(6) $$V_{out}=V_{in}\sin\left(\omega_{0}t+\theta \right)\times \;V_{ref}\sin\left(\omega_{0}t\right)\;\;\;\;\;\;\;\;\;\;\;\;\;\;\;=\frac{1}{2}V_{in}V_{ref}cos\theta -\frac{1}{2}V_{in}V_{ref}\cos\left({2\omega }_{0}t+\theta \right).$$

**Figure 12:**
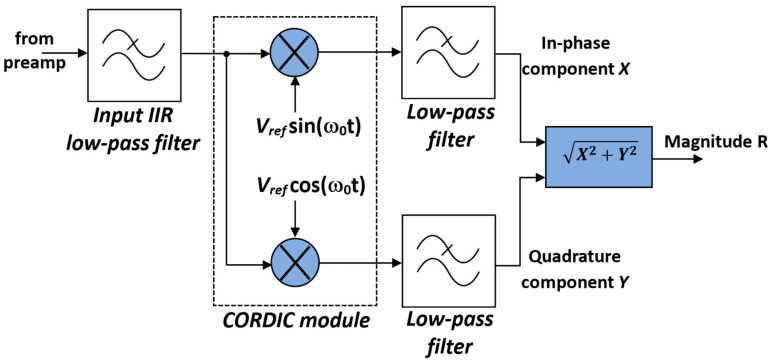
Basic architecture of the lock-in amplifier.

A narrow-band low-pass filter removes the high-frequency term, leaving only the DC output, proportional to the amplitude of the input signal $V_{in}\;$ and to the cosine of the phase difference (in-phase component, X). To eliminate the dependence on the phase θ, a second multiplication with a 90°-shifted reference $V_{ref}\cos\left(\omega_{0}t\right)\;$ yields the quadrature component Y. These two outputs form complex vectors, which are sent from the LIA to the microcontroller, where signal amplitude $R=\sqrt{X^{2}+Y^{2}}$ and phase $\theta =\;{tan}^{-1}(Y/X)$ are computed. The basic architecture of this dual-phase LIA is reported in Figure 12.

The input low-pass IIR filter in Figure 12, which processes the digital output signal of the preamplifier board, limits the bandwidth BW of the digitized QTF signal to approximately 20 kHz, which is abundantly sufficient, since the resonant frequency of the QTFs usually employed is included in this bandwidth. This input filter reduces the inband quantization noise, thus enhancing the signal-to-noise ratio (SNR). Assuming an ideal 14-bit ADC with quantization noise as the dominant error source and considering the oversampling ratio fs/(2·BW) of about 5.6, the theoretical SNR improvement is $10\cdot \log_{10}\left(5.6\right)≅7.5\;dB$.

**Figure 13:**
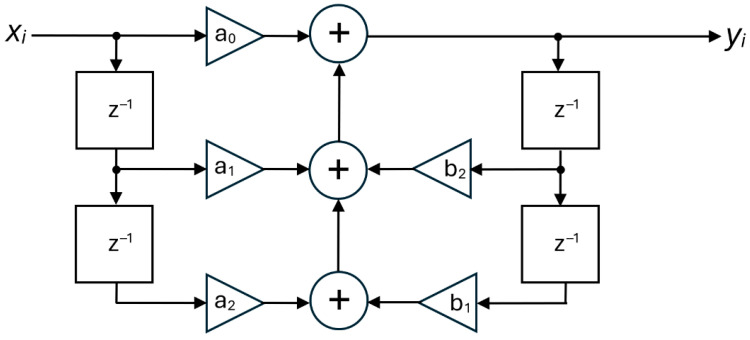
Recursive architecture of one of the biquad sections of the sixth order, type II Chebyshev filter.

The input filter has a complex, three-stage architecture. The first stage is a simple moving average filter, implemented with a first-order cascaded integrator–comb (CIC) structure, which also performs decimation by a factor of 32. The second stage is a fifth-order shaping filter, useful for improving the transition band characteristics, and the third stage is a sixth-order, type II Chebyshev filter. In particular, the last stage is composed of three cascaded biquad sections, each realized according to the classic second-order recursive linear filter architecture, shown in Figure 13.

**Figure 14:**
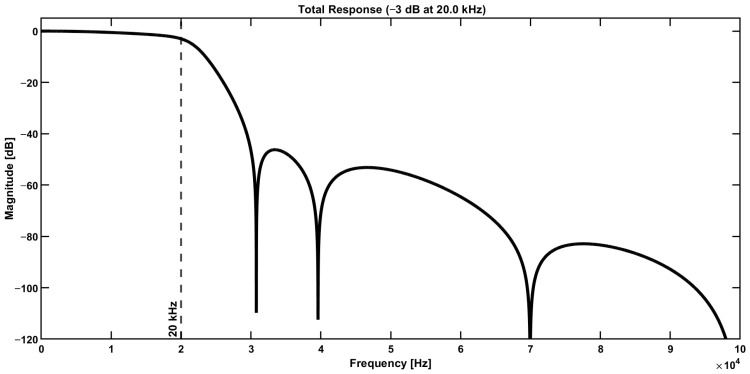
Frequency response of the low-pass input filter of Figure 12.

The frequency response of the input low-pass filter is illustrated in Figure 14.

Instead of using complex and resource-demanding hardware multipliers to obtain the products between the filtered input signal and the two reference signals, a CORDIC module was used to generate both $V_{ref}\cos\left(\omega_{0}t\right)\;$ and $V_{ref}\sin\left(\omega_{0}t\right)\;$ and to perform the multiplications at the same time. In fact, if we assign to the initial value of the vector $p_{0}$ the value [$x_{i},0]$, where $x_{i}$ is the sample of the filtered input signal to be multiplied by the sample of the sine wave reference, the result of the CORDIC algorithm is directly proportional to the product of the two samples. Thus, effective integration between sine wave generation and multiplication allows sparing FPGA hardware resources, which can be employed to increase the complexity and the selectivity of the filters, improving SNR performance.

**Figure 15:**
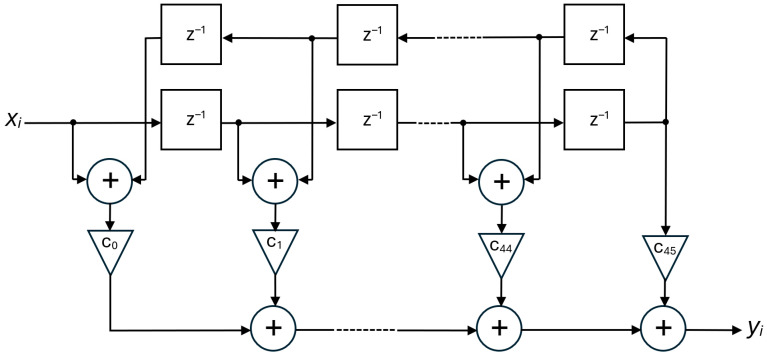
Folded structure of the FIR filter.

As far as the low-pass output filters in Figure 12 are concerned, they are composed of two cascaded stages. The first one has the same structure as the input IIR filter previously described, whereas the second one is a 90-tap FIR filter with a folded structure, schematically depicted in Figure 15, which reduces the number of complex multiplications by about 50%.

The frequency response of the LIA output filter can be controlled by varying the sampling times of the different sections of the circuit. As a result, the LIA integration time can assume eight different values distributed as powers of 2, ranging from 7.5 ms to 960 ms.

## 4. Experimental Results

**Figure 16:**
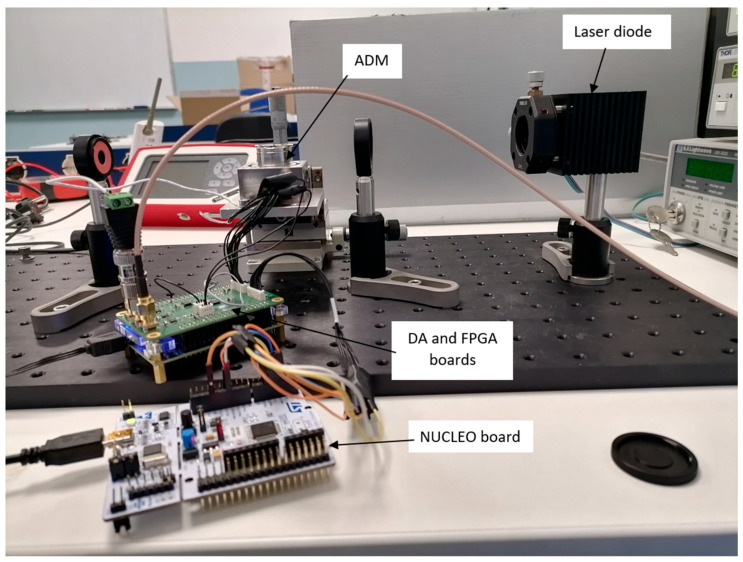
QEPAS sensor for H_2_O detection: experimental setup.

As mentioned in the Introduction, to validate the full functionality of the electronic blocks described above inside a real QEPAS sensor, an experimental test bench was set up for the detection of water vapor (H_2_O) in open air. The experimental setup is illustrated in Figure 16.

The laser source used is a 1392 nm DFB laser diode from Nanoplus. The preamplifier, configured as a charge amplifier with $C_{f}=2.2\;pF,R_{f}=100\;M\Omega$, and $R_{g}=0\;\Omega$, is assembled into the ADM and is not visible in the picture shown in Figure 16. The FPGA provides the modulation signal to the laser driver, which is a standard lab instrument from Thorlabs, whereas temperature control of the laser source is managed by a Thorlabs TEC controller. As mentioned above, system configuration, including control of the preamplifier and FPGA, and data exchange are managed by means of the STM32 NUCLEO board, interfaced with a custom Python 3.11 GUI running on a PC.

In characterization mode, this Python GUI is used to set the programmable parameters of the excitation sine wave, such as starting frequency, number of frequency points, frequency step with a resolution of 11.6 mHz, and amplitude, which can be conveniently controlled in order to avoid saturation of the signal processing chain. The response of the QTF, measured by means of the lock-in amplifier, is transmitted via SPI interface to the NUCLEO board, and for the QTF used in this experiment, the extracted value of the resonant frequency is $f_{0}=15,158.2\;Hz$, whereas the Q-factor is approximately 15,000.

**Figure 17:**
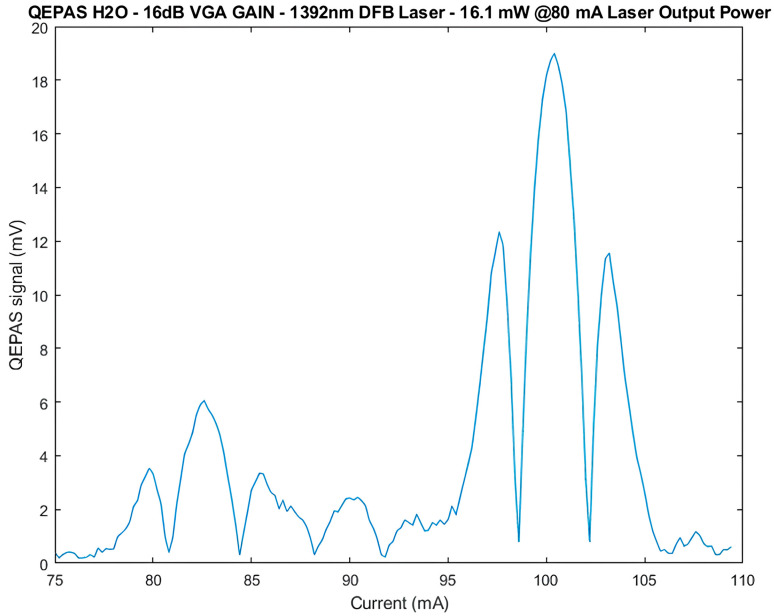
Amplitude R of the QEPAS signal (mV) as a function of the laser current (mA).

For the first tests, the QEPAS sensor was used in scan mode, the laser current was swept from 75 mA to 110 mA with steps of 1 mA, and the VGA gain was set to 16 dB. In Figure 17, the measured value of the amplitude $R=\sqrt{X^{2}+Y^{2}}$ of the QEPAS signal is reported as a function of the laser current. The signal exhibits the typical expected spectral shape for H_2_O, with a well-defined main lobe corresponding to the absorption peak, confirming the correct operation of the preamplifier, lock-in amplifier, function generator, and DA board.

The estimated signal-to-noise ratio, obtained with the lock-in amplifier integration time set to its maximum value of 960 ms, is about 40 dB. This value was obtained considering 199 measurements carried out in line-locking mode, i.e., with the laser current set at a fixed value of about 100 mA, corresponding to the peak of the main lobe of the QEPAS signal in Figure 17. The SNR was evaluated as the ratio between the average value of these measurements and their standard deviation. This represents a good result considering the low optical power of the laser, approximately 16 mW. Notably, increasing the VGA gain has no significant effects on the SNR, indicating that the dominant noise source lies at the input stage and that the system is operating close to its intrinsic noise floor.

**Figure 18:**
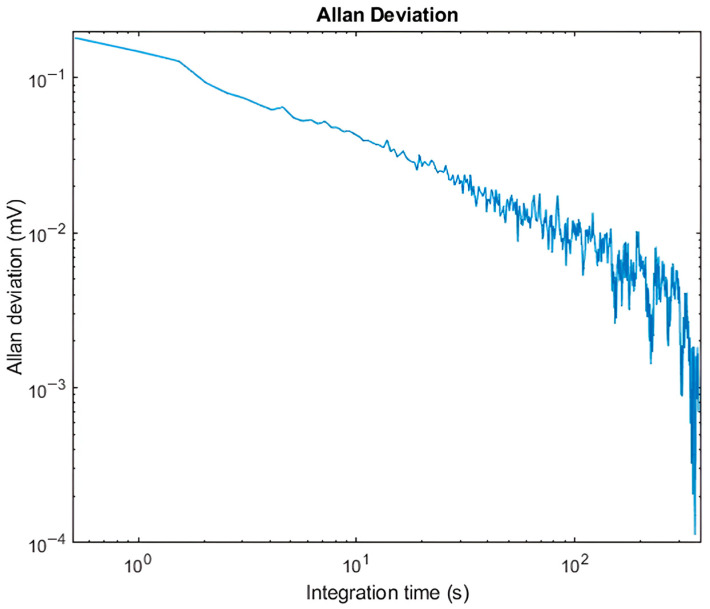
Allan deviation plot for the QEPAS sensor as a function of the LIA integration time τ.

The Allan deviation for the QEPAS sensor was also measured in order to evaluate the stability of the system over time and is represented in Figure 18, which reports the noise level in mV as a function of the integration time τ. Results for long values of the integration time were obtained by averaging output samples obtained using its maximum allowed value. A longer τ results in improved noise suppression, and the measured 1/√τ behavior results are consistent with the one expected for an averaged white noise. Measurements were performed with the laser turned on and frequency-locked far from the H_2_O absorption line, ensuring that no absorption signal contributed to the output.

Since the plot exhibits the expected 1/√τ trend, this indicates that the system noise performance is dominated by thermal noise sources [23]. In this case, the primary contribution is attributed to the thermal noise associated with the resistor R_p_ in the electric model of the QTF, shown in Figure 3, which sets the fundamental sensitivity limit of the sensor under these conditions.

**Figure 19:**
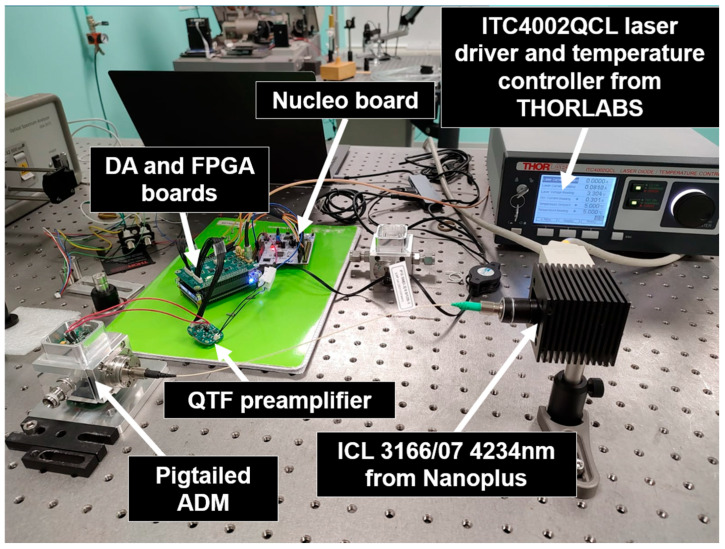
Experimental setup for the detection of CO_2_.

Additional tests were carried out using a different experimental setup. In this case, an ICL 3166/07 interband cascade laser, from Nanoplus, Meiningen, Germany, operating at 4234 nm was employed to target CO_2_ in ambient air, and a pigtailed laser source and ADM were used. Unlike the previous setup, our preamplifier was not integrated into the ADM. In fact, the ADM was already equipped with a built-in preamplifier, which was not used. Instead, the QTF terminals were connected to the inputs of our circuit externally to the ADM by means of cables, as illustrated in Figure 19. Although this arrangement is sub-optimal in terms of signal-to-noise ratio, the primary objective was to validate the complete functionality of the latest version of the electronics in a real QEPAS environment under modified operating conditions. Concerning the laser driver and the temperature controller, an ITC4002QCL laboratory instrument, from Thorlabs, Newton, NJ, USA, was used, and to reduce its noise contributions to negligible levels, the noise reduction filter available internally was applied.

**Figure 20:**
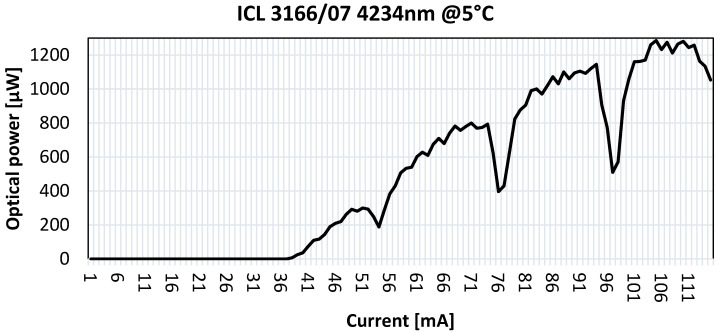
Optical power as a function of laser current, with the laser temperature stabilized at 5 °C.

The optical output power of the laser was characterized as a function of the driving current, and the laser temperature was stabilized at 5 °C. As shown in Figure 20, the maximum optical power measured was 1200 µW at 110 mA.

**Figure 21:**
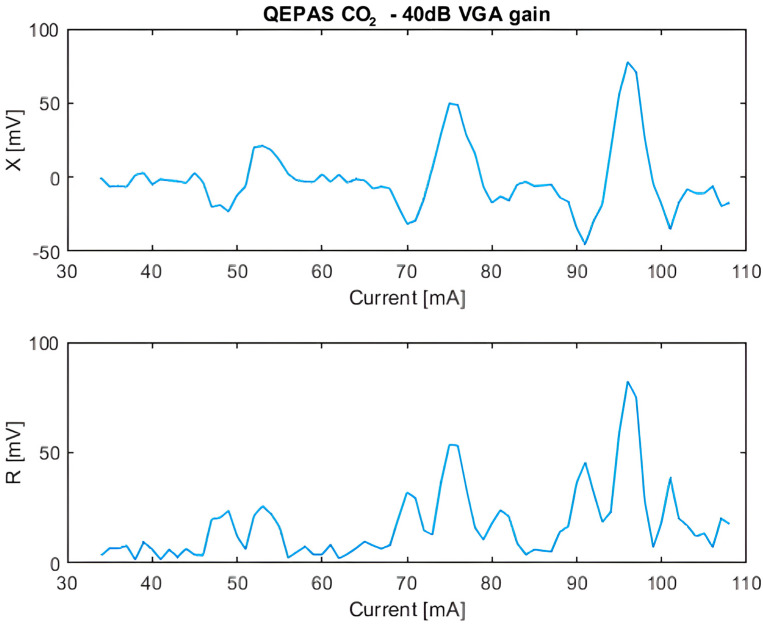
In-phase X and amplitude R components of the QEPAS signal for ambient air CO_2_, obtained in scan mode.

The response of the QEPAS sensor to ambient CO_2_ was measured. Figure 21 shows both the in-phase component X and the signal amplitude R obtained in scan mode. For this measurement, the lock-in amplifier integration time was set to its maximum value of 960 ms, and the total transimpedance gain of the preamplifier, always configured as a charge amplifier, was set to $11.7\times 10^{3}\;M\Omega$, corresponding to a VGA gain of 40 dB.

At a laser current value of 75.6 mA, the measured optical power was approximately 400 µW. Under these conditions, an SNR of 25.4 dB was achieved, which is an appreciable result considering the non-ideal mechanical assembly and the moderate optical power available. To assess the impact of amplification on signal quality, the VGA gain was varied in line-locking mode, but no significant change in the SNR was observed. This confirms the robustness of the preamplifier design, which effectively maintains performance close to the noise floor of the system.

In scan mode, current step sizes of 0.1 mA, 0.5 mA, and 1 mA were considered, and 1 mA steps were proven sufficient for accurate signal reconstruction, allowing for faster acquisition without loss of resolution.

**Figure 22:**
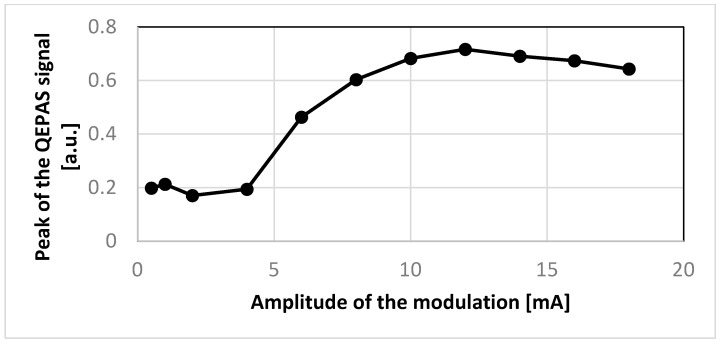
Amplitude of the main peak of the QEPAS signal as a function of the laser modulation amplitude in line-locking mode.

Finally, the behavior of the amplitude R of the QEPAS signal was studied as a function of the laser modulation amplitude. For this purpose, the QEPAS sensor was operated in line-locking mode, with a fixed value of the laser current set at the main peak of the QEPAS signal obtained in scan mode, i.e., 75.6 mA. As shown in Figure 22, the experimental data shows that the amplitude of the QEPAS signal increases with the modulation index, reaches a maximum, and finally starts decreasing. This closely follows the theoretical expectations [24,25], confirming the proper modulation behavior of the laser and the full functionality of the QEPAS sensor based on the designed electronics.

## 5. Discussion and Conclusions

A compact electronic system including the main building blocks needed to operate a QEPAS sensor has been designed and verified. The interface circuit for the QTF is a front-end configurable as charge or transimpedance amplifier, followed by a variable gain voltage amplifier. The whole analog chain is fully differential to eliminate the common-mode noise, and the transimpedance gain is programmable by means of a digital word to enhance flexibility and adapt the circuit to different applications. The output signal is converted into digital form, and a phase-sensitive detection scheme is applied on it by means of a lock-in amplifier implemented on an FPGA board. To spare hardware resources, which can be dedicated to implement high-performance filters with the purpose of increasing SNR, the CORDIC algorithm has been exploited to generate the samples of the LIA reference signal and to multiply them with the samples of the QTF signal, without recourse to hardware multipliers. CORDIC has also been conveniently applied for the generation of the sine wave needed for modulating the laser beam intensity. A custom board, capable of managing three laser drivers, has also been realized for converting into analog form and filtering the samples of the laser modulation signal. A real QEPAS sensor has been built, based on the designed electronic blocks, and some experiments have been carried out to verify its full functionality. The system successfully extracted clear QEPAS signals for both environmental H_2_O and CO_2_, confirming the proper operation of all the components. A signal-to-noise ratio of about 40 dB was measured in the detection of H_2_O and 25.4 dB for the CO_2_ case despite the very low power of the employed laser sources, especially for the CO_2_ measurements. Noise analysis using Allan deviation confirmed that the system residual noise follows the expected 1/√τ behavior, consistent with white noise being filtered by the lock-in amplifier. This validates the system in terms of capability for stable long-term measurements.

On the one hand, the development of compact and effective electronic building blocks for QEPAS sensors will allow the possible application of this detection technique to a broader range of situations, such as, for instance, installation of the sensor on board a drone. On the other hand, the decrease in the costs and size of the electronics will contribute to the further spread gas-sensing systems based on the QEPAS technique, making it more appealing to a larger set of possible users in both industrial and scientific applications. From this perspective, the goal of future developments is the integration on a single, compact board of the described blocks together with a laser driver and a TEC controller.

## Data Availability

The original contributions presented in this study are included in the article. Further inquiries can be directed to the corresponding author.

## Abbreviations

The following abbreviations are used in this manuscript:

ADC	Analog-to-Digital Converter
ADM	Acoustic Detection Module
CORDIC	COordinate Rotation DIgital Computer
DAC	Digital-to-Analog Converter
DC	Direct Current
FPGA	Field Programmable Gate Array
GUI	Graphic User Interface
ICL	Interband Cascade Laser
LIA	Lock-In Amplifier
OPAMP	Operational Amplifier
QEPAS	Quartz-enhanced Photoacoustic Spectroscopy
QTF	Quartz Tuning Fork
SNR	Signal-to-Noise Ratio
SPI	Serial Peripheral Interface
TEC	Thermoelectric Cooler
TIA	Transimpedance Amplifier
VGA	Variable Gain Amplifier
